# N-myc downstream-regulated gene 2 expression is associated with glucose transport and correlated with prognosis in breast carcinoma

**DOI:** 10.1186/bcr3628

**Published:** 2014-03-18

**Authors:** Ji Ma, Wenchao Liu, Hang Guo, Shaoqing Li, Wei Cao, Xilin Du, Shixiong Lei, Wugang Hou, Lize Xiong, Libo Yao, Nanlin Li, Yan Li

**Affiliations:** 1 The State Key Laboratory of Cancer Biology and Department of Biochemistry and Molecular Biology, The Fourth Military Medical University, 169 Changle West Rd, Xi’an 710032, China; 2 Department of Oncology, Xijing Hospital, The Fourth Military Medical University, 127 Changle West Rd, Xi’an 710032, China; 3 Department of Breast Surgery, Lanzhou General Hospital of the People’s Liberation Army, 333 Binhe South Rd, Lanzhou 730000, China; 4 Department of Oral Biology, Stomatology School, The Fourth Military Medical University, 145 Changle West Rd, Xi’an 710032, China; 5 Department of Interventional Radiology, Tangdu Hospital, The Fourth Military Medical University, 1 Xinsi Rd, Xi’an 710032, China; 6 Department of General Surgery, Tangdu Hospital, The Fourth Military Medical University, 1 Xinsi Rd, Xi’an 710032, China; 7 Department of Anesthesiology, Xijing Hospital, The Fourth Military Medical University, 127 Changle West Rd, Xi’an 710032, China; 8 Department of Vascular and Endocrine Surgery, Xijing Hospital, The Fourth Military Medical University, 127 Changle West Rd, Xi’an 710032, China

## Abstract

**Introduction:**

N-myc downstream-regulated gene 2 (*NDRG2*), a novel tumour suppressor and cell stress-related gene, is involved in many cell metabolic processes, such as hormone, ion and fluid metabolism. We investigated whether *NDRG2* is involved in any glucose-dependent energy metabolism, as well as the nature of its correlation with breast carcinoma.

**Methods:**

The correlations between NDRG2 expression and glucose transporter 1 (GLUT1) expression in clinical breast carcinoma tissues were analysed. The effects of NDRG2 on glucose uptake were assessed in breast cancer cells and xenograft tumours. The consequences of NDRG2-induced regulation of GLUT1 at the transcription and translation levels and the interaction between NDRG2 and GLUT1 were examined.

**Results:**

Data derived from clinical breast carcinoma specimens revealed that (1) patients with high NDRG2 expression had better disease-free survival and overall survival than those with low NDRG2 expression and (2) NDRG2 expression was negatively correlated with GLUT1 expression in these breast carcinoma tissues. NDRG2 inhibited glucose uptake by promoting GLUT1 protein degradation without affecting *GLUT1* transcription in both breast cancer cells and xenograft tumours. In addition, NDRG2 protein interacted and partly colocalised with GLUT1 protein in cell cytoplasm areas.

**Conclusions:**

The results of our study support the notion that NDRG2 plays an important role in tumour glucose metabolism, in which GLUT1 is a likely candidate contributor to glucose uptake suppression and tumour growth. Targeting the actions of NDRG2 in cell glucose-dependent energy delivery may provide an attractive strategy for therapeutic intervention in human breast carcinoma.

## Introduction

N-myc downstream-regulated gene 2 (*NDRG2*) is a member of the *NDRG* family [1] and was first identified and cloned in our laboratory from a normal human brain cDNA library by subtractive hybridisation [2]. Accumulating evidence indicates that *NDRG2* is a tumour suppressor gene that is downregulated or undetectable in many human cancers [1,3]. The overexpression of NDRG2 is able to enhance cell apoptosis, inhibit cell proliferation and suppress angiogenesis in many malignant tumours [4,5]. Recently, researchers showed that NDRG2 expression was inversely associated with TNM (tumour, node, metastasis) stage in 189 breast carcinoma tissues and paired normal breast tissues [6].

In addition to its known antitumoural function, *NDRG2* may be a metabolism-related gene regulated by many hormones, including adrenal steroids [7], dexamethasone [8,9], insulin [10-12], androgen [13], oestrogen [14] and aldosterone [15]. NDRG2 was found to act as a regulator of myoblast proliferation and can be regulated by anabolic and catabolic factors [8]. In skeletal muscle, NDRG2 is a substrate for several serine-threonine protein kinases, including protein kinase B (Akt) and serum- and glucocorticoid-induced kinase 1 (SGK1) [10,16]. NDRG2 was also found to induce amiloride-sensitive Na^+^ transport in *Xenopus laevis* oocytes and Fischer rat thyroid cells [17]. In a previous study, we found that NDRG2 promoted Na^+^/K^+^-ATPase activity to promote cell Na^+^ transport and fluid balance [14]. We also identified that NDRG2 acted as a key molecule in pancreatic *β* cells and was involved in Akt-mediated protection of *β* cells against lipotoxicity [11]. The evidence described herein suggests that *NDRG2* is a metabolism-related gene and plays important roles in cellular physiological metabolism. Furthermore, NDRG2 was recently shown to respond to cellular stress under a series of environmental stress conditions [1]. However, very little information is available regarding the function of NDRG2 in tumour metabolism.

Mammalian cells depend on glucose as a major substrate for energy production [18]. Warburg showed that tumour cells could metabolise many orders of magnitude larger amounts of glucose than their differentiated normal counterparts [19,20]. The transport of glucose across the plasma membrane is the first rate-limiting step for glucose metabolism and is mediated via glucose transporter proteins (GLUTs) [18]. At present, 14 members of the GLUT family have been identified [21]. GLUT1 is broadly expressed in the body tissues and is involved in glucose uptake in the basic state. Elevated levels of GLUT1 have been shown to be present in many human cancers, including head and neck, breast, lung and ovarian [22,23]. Moreover, several reports have suggested that GLUT1 represents potential regulatory targets of oncogenes or tumour suppressors [24-26].

We posited the following questions: (1) whether NDRG2 expression is associated with any GLUT expression, as well as the nature of its correlation with breast carcinoma; (2) whether and why NDRG2 affects the glucose uptake; (3) what would be the significance of the interactions between NDRG2 and the GLUTs; and (4) whether this regulation of NDRG2 on the GLUTs exists *in vivo*. In our present study, we tested the hypothesis that a possible mechanism of NDRG2 induces its participation in cancer cell energy metabolism through the regulation of GLUTs in breast carcinoma.

## Methods

### Tissue samples and study cohort

This study was approved by the Ethics Committee of the Fourth Military Medical University. All patients from whom we obtained the 30 pairs of breast carcinoma and adjacent normal breast tissue specimens, as well as the 269 breast carcinoma sample study cohort, provided their full consent to participate in the study at the Xijing Hospital of the Fourth Military Medical University (Xi’an, China). NDRG2 and GLUT1 expression were detected in all specimens. Tissue specimens were examined separately by two pathologists under double-blinded conditions without prior knowledge of the clinical status of the specimens.

### Immunohistochemistry detection

Immunohistochemistry (IHC) was performed using the avidin-biotin-peroxidase complex method on all breast carcinoma samples. All sections were deparaffinised in xylenes and dehydrated through a gradient concentration of alcohol before endogenous peroxidase activity was blocked using 0.5% H_2_O_2_ in methanol for 10 minutes. After nonspecific binding was blocked, the slides were incubated with NDRG2 antibody (1:200; Abnova, Taipei, Taiwan) or GLUT1 antibody (1:200; Santa Cruz Biotechnology, Santa Cruz, CA, USA) in phosphate-buffered saline (PBS) at 4°C overnight in a humidified container. Biotinylated goat anti-rabbit immunoglobulin G (IgG) (1:400; Sigma-Aldrich, St Louis, MO, USA) was incubated with the sections for 1 hour at room temperature and detected using a streptavidin-peroxidase complex. The brown colour indicative of peroxidase activity was developed by incubation with 0.1% 3,3′-diaminobenzidine (Sigma-Aldrich) in PBS with 0.05% H_2_O_2_ for 5 minutes at room temperature. The appropriate positive and negative controls were included in each run of IHC.

### Staining evaluation

An immunoreactivity score system based on the proportion and intensity of positively stained cancer cells was applied. The two extensional standards taken were as follows: (1) the number of positively stained cells ≤5%, scored 0; 6% to 25%, scored 1; 26% to 50%, scored 2; 51% to 75%, scored 3; and >75%, scored 4; and (2) the intensity of stain colourless, scored 0; pallideflavens, scored 1; yellow, scored 2; and brown, scored 3. Extensional standards (1) and (2) were multiplied, and the staining grade was stratified as absent (score 0), weak (score 1 to 4), moderate (score 5 to 8) or strong (score 9 to 12). Specimens were rescored if the difference of scores from the two pathologists was greater than 3. Tumours with moderate or strong immunostaining were classified as having high expression, and tumours with absent or weak immunostaining were classified as having low expression.

### Cell cultures and reagents

T-47D and SK-BR-3 cells were obtained from the American Type Culture Collection (Manassas, VA, USA) and cultured in a humidified incubator under 5% CO_2_ in Dulbecco’s modified Eagle’s medium (DMEM) (Invitrogen/Life Technologies, Carlsbad, CA, USA) supplemented with 10% foetal bovine serum (FBS) and 2 mM L-glutamine. NDRG2 antibody was purchased from Abnova. GLUT1, hemagglutinin (HA), actin, flag and tubulin antibodies were obtained from Santa Cruz Biotechnology.

### Gene transfection

The cells (1 × 10^6^ cells/well) were seeded into six-well plates and transfected with the following constructs using Lipofectamine 2000 (Invitrogen/Life Sciences) according to the manufacturer’s instructions as follows: NDRG2 expression plasmid (pCMV-flag-NDRG2), GLUT1 expression plasmid (pCMV-eGFP-GLUT1), HA-ubiquitin or small interfering RNA (siRNA) that targeted *NDRG2*. The target sequences of NDRG2 siRNA and control siRNA are given in Additional file 1: Table S1.

### Gene infection

Cells were seeded into six-well plates at a density of 1 × 10^6^ cells/well and incubated to reach approximately 80% confluence. After the medium was removed, adenovirus expressing NDRG2 (Ad-NDRG2) or the negative control adenovirus expressing LacZ (Ad-LacZ) was added to serum-free DMEM, incubated for 2 hours, replaced with fresh DMEM supplemented with 10% FBS and incubated for another 48 hours. Recombinant adenoviruses carrying NDRG2 or LacZ were purchased from Benyuan Zhengyang Gene Technology Company (Beijing, China).

### Immunoblotting

Both cells and breast tissues were lysed in radioimmunoprecipitation assay buffer (0.05 M Tris-HCl (pH 7.4), 0.15 M NaCl, 0.25% deoxycholic acid, 1% Nonidet P-40, 1 mM ethylenediaminetetraacetic acid (EDTA), 1 mM phenylmethylsulfonyl fluoride, 10 mg/ml aprotinin and 10 mg/ml leupeptin). Protein concentrations were measured using a bicinchoninic acid protein assay (Pierce Biotechnology, Rockford, IL, USA). Proteins were resolved by SDS-PAGE and transferred to Hybond enhanced chemiluminescence nitrocellulose membranes (Amersham Biosciences, Piscataway, NJ, USA). The blots were probed with the different primary antibodies and species-matched secondary antibodies. The bands were detected using enhanced chemiluminescence (Pierce Biotechnology) or the Odyssey Imaging System (LI-COR Biosciences, Lincoln, NE, USA).

### Real-time PCR

The RNA extracted from cells with TRIzol reagent (Invitrogen/Life Technologies, Carlsbad, CA, USA) was converted to combinational cDNA with the RevertAid First Strand cDNA Synthesis Kit (Fermentas/Thermo Scientific; Pittsburgh, PA, USA). Real-time PCR analysis was performed using the Prism 7500 Real-Time PCR System (Applied Biosystems, Foster City, CA, USA) and the SYBR *Premix Ex Taq* II (Tli RNase H Plus) kit (TaKaRa Bio, Shiga, Japan) according to the manufacturer’s instructions. The relative gene expression levels were calculated using the 2^-ΔΔCt^ method, in which Ct represented the threshold cycle and β-actin was used as a reference gene. The primer sequence is given in Additional file 1: Table S1.

### Cell proliferation assay

Cell growth following transfection was evaluated by 3-(4,5-dimethylthiazol-2-yl)-2,5-diphenyltetrazolium bromide (MTT) assay. Cells were seeded into a 96-well plate (1 × 10^4^ cells per well) and incubated for 24 hours. The cells were then incubated with 0.5 mg/ml MTT (Sigma-Aldrich). Four hours later, the medium was replaced with 100 μl of dimethyl sulfoxide (Sigma-Aldrich) and vortexed for 10 minutes. Absorbance was then recorded at 490 nm using an Easy Reader 340 AT plate reader (SLT-Lab Instruments, Salzburg, Austria). Relative values of optical density were calculated as a percentage of the control. All experiments were performed three times independently.

### Glucose uptake assay

Prior to being harvested, adherent cultures of control and NDRG2 adenovirus- or siRNA-treated cells in DMEM containing 25 mM glucose were washed twice with cold PBS and then lysed with ion-free H_2_O for 5 minutes on ice. The glucose content was measured with a D-glucose measurement kit (GAHK-20; Sigma-Aldrich) according to the manufacturer’s protocol.

### Immunofluorescence assay

Cells were fixed in a freshly prepared solution of 4% paraformaldehyde, rinsed and permeabilised with 0.1% Triton X-100 in PBS. Permeabilised cells were then incubated with horse serum in PBS to block nonspecific binding. After being washed with PBS, the cells were incubated overnight at 4°C with mouse anti-NDRG2 antibody (diluted 1:150), rabbit anti-GLUT1 antibody (diluted 1:150) and fluorescein isothiocyanate (FITC)-conjugated anti-mouse antibody (diluted 1:400; Sigma-Aldrich) or cyanine 3 (Cy3)-conjugated anti-rabbit antibody (diluted 1:400; Sigma-Aldrich). The isotype mouse and rabbit IgGs were used as negative controls. Dual-colour detection was performed using a laser confocal microscope after treatment with 4′,6-diamidino-2-phenylindole (DAPI) to label nuclear DNA.

### Immunoprecipitation

Transfected or untransfected cells were incubated with 1 ml of lysis buffer containing 150 mM NaCl, 50 mM Tris-HCl (pH 7.4), 1% Lubrol (polyethylene glycolmonocetyl ether; MP Biomedicals, Solon, OH, USA) and 5 mM EDTA, as well as protease inhibitors, for 30 minutes at 4°C. The insoluble fraction was eliminated through centrifugation at 10,000 × *g* for 30 min at 4°C. After centrifugation, the lysates were incubated with the antibody of interest, and protein A or G was conjugated to sepharose (Pierce Biotechnology) for 8 hours at 4°C. To quantify the total amount of protein loaded, 20 μl of the lysates was saved. Beads were washed four times with lysis buffer. Proteins were eluted in SDS-PAGE sample buffer and separated by SDS-PAGE for immunoblot analysis. The blots were then probed with peroxidase-conjugated goat anti-mouse or goat anti-rabbit antibodies and visualised by using an enhanced chemiluminescence reagent (Pierce Biotechnology).

### Xenograft study in nude mice

For inoculation into nude mice, SK-BR-3 or MDA-MB-231 cells were washed with PBS, digested with trypsin and resuspended in serum-free DMEM. After centrifugation (800 rpm), the cell pellets were resuspended in DMEM. The cell suspension (1 × 10^6^ cells in a 100-ml volume of PBS) was injected subcutaneously into the hind legs of 4-week-old female BALB/c athymic (*nu*/*nu*) mice (SLAC Laboratory Animal Company, Shanghai, China). When the tumours reached a volume of approximately 200 mm^3^, the mice were arbitrarily assigned to different groups (*n* = 6 each) to receive intratumoural injections of 0.5, 1 or 2 × 10^9^ plaque-forming units (PFU) of Ad-NDRG2, 2 × 10^9^ Ad-LacZ or PBS. Intratumoural injections were repeated every 3 days for a total of 21 days. Tumours were measured (perpendicular diameters) every 3 days, and their volumes were calculated. On day 21, the mice were killed and their tumours were removed for analysis. Tumour volumes were calculated based on caliper measurements of the length and width of the lesions using the following formula: 0.5 × length × (width^2^). The tumour growth curve was then derived from these data.

All of the experimental procedures were conducted in accordance with the Detailed Rules for the Administration of Animal Experiments for Medical Research Purposes issued by the Ministry of Health of China and received ethical approval by the Animal Experiment Administration Committee of the Fourth Military Medical University (Xi’an, China). All efforts were made to minimise the animals’ suffering and reduce the number of animals used.

### Statistical analysis

*In vitro* experiments were performed three times, and each experiment was performed in triplicate. Data from all quantitative assays are expressed as the means ± SD and were analysed statistically using one-way analysis of variance, independent samples *t*-test or Student’s *t*-test. In the clinical specimens study, the associations between NDRG2 expression and categorical variables were analysed using the χ^2^ test or Fisher’s exact test as appropriate. Correlations between NDRG2 expression and the expression of other molecules were analysed by using the Spearman correlation test. Kaplan–Meier analysis was used to evaluate disease-free survival and overall survival. *P* < 0.05 was considered statistically significant.

## Results

### Relationship between NDRG2 expression and clinical histopathological characteristics in breast carcinoma

**Figure 1:**
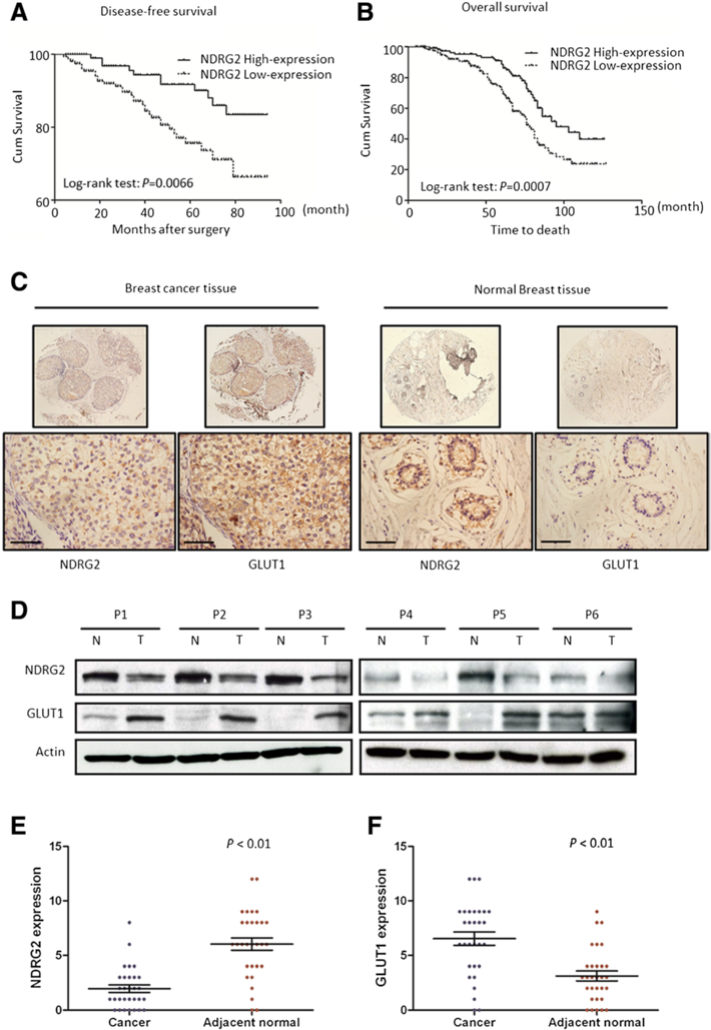
**NDRG2 is correlated with increased survival and negatively correlated with GLUT1 in breast carcinoma.** Kaplan–Meier analysis was carried out according to N-myc downstream-regulated gene 2 (NDRG2) expression levels of disease-free survival **(A)** and overall survival **(B)**. **(C)** Serial immunostained sections for NDRG2 and glucose transporter 1 (GLUT1) in breast cancer and normal tissues were analysed. Original magnification, 40× (top) and 400× (bottom); scale bars = 50 μm. **(D)** Protein was extracted from matched breast tumour tissue (T) and adjacent normal tissue (N) and subjected to immunoblot analysis to examine NDRG2 and GLUT1 expression. β-actin served as a loading control. P: patient. Relative expression levels of NDRG2 **(E)** and GLUT1 **(F)** in human breast cancer and adjacent normal tissue are shown. immunoreactivity score distribution of cancer and adjacent normal tissue were represented with black and brown closed circles, respectively. The horizontal lines presented are means; error bars represented SD from 30 samples. *P* < 0.01 was considered a statistically significant difference.

To assess the significance of NDRG2 protein expression in the development and progression of breast cancer, we compared the histopathological characteristics of 269 breast cancer samples with available NDRG2 protein status. The correlations between NDRG2 expression and different clinical histopathological factors are presented in Table 1. Significant correlations were found between low NDRG2 expression and advanced TNM staging (*P* < 0.0001), high proliferation index (Ki67 status; *P* = 0.006), positive human epidermal growth factor receptor 2 (HER2) status (*P* = 0.010) and poor histological differentiation (*P* < 0.0001). However, high NDRG2 expression was correlated with positive oestrogen receptor (ER) status (*P* = 0.010). Correlation coefficients are presented in Table 2. Furthermore, Kaplan–Meier analysis was used to evaluate the disease-free survival and overall survival of patients with breast cancer and NDRG2 protein expression. The results show that patients with high NDRG2 expression in breast tumour tissues had better disease-free survival than those with low NDRG2 expression (*P* = 0.0066 by logrank test) (Figure 1A). Breast cancer patients with low NDRG2 expression had a higher risk of relapse than those with high NDRG2 expression. A statistically significant association between long overall survival and high NDRG2 protein levels was found in breast cancer patients. Breast cancer patients with high NDRG2 expression had longer overall survival than patients with low NDRG2 expression *P* = 0.0007 by logrank test) (Figure 1B).

**Table 1:** Statistical results of NDRG2 expression in 269 breast cancer specimens

**Variables**	***N***	**NDRG2 expression**		
		**Low expression, *n *** **(%)**	**High expression, *n *** **(%)**	***P-value***
Age (years)				0.104^b^
≤50	112	72 (64.3)	40 (35.7)	
>50	157	85 (54.1)	72 (45.9)	
Tumour size				0.536^b^
≤2 cm	121	68 (56.2)	53 (43.8)	
>2 cm	148	89 (60.1)	59 (39.9)	
TNM stage				<0.0001^b^
I to II	114	48 (42.1)	66 (57.9)	
III to IV	155	109 (70.3)	46 (29.7)	
Lymph node metastasis				0.009^b^
Negative	114	56 (49.1)	58 (50.9)	
Positive	155	101 (65.2)	54 (34.8)	
Histology				<0.0001^c^
Poorly differentiated	103	75 (72.8)	28 (27.2)	
Moderately differentiated	83	52 (62.7)	31 (37.3)	
Well differentiated	83	30 (36.1)	53 (63.9)	
Tumour invasion				0.387^b^
No	134	82 (61.2)	52 (38.8)	
Yes	135	75 (55.6)	60 (44.4)	
ER status				0.010^b^
Negative	79	56 (70.9)	23 (29.1)	
Positive	190	101 (53.2)	89 (46.8)	
Her-2 status				0.010^b^
Negative	201	108 (53.7)	93 (46.3)	
Positive	68	49 (72.1)	19 (27.9)	
Ki67 status				0.006^b^
Negative	119	58 (48.7)	61 (51.3)	
Positive	150	99 (66.0)	51 (34.0)	

^a^ER, Oestrogen receptor; Her-2, Human epidermal growth factor receptor 2; NDRG2, N-myc downstream-regulated gene 2; TNM, tumour, node, metastasis. ^b^Fisher’s exact test was used for statistical analyses. *P* < 0.05 were considered statistically significant. ^c^Pearson’s χ^2^ test was used for statistical analyses.

**Table 2:** **Correlation of NDRG2 expression with clinical histopathologic characteristics in 269 breast cancer specimens**^**a**^

**Variables**	**NDRG2 expression**	
	**Correlation coefficient (*r***_***s***_ ***)***	***P*-value**
Age (years)	0.101	0.097
Tumour size	-0.040	0.516
TNM stage	-0.283	0.0001
Lymph node metastasis	-0.161	0.008
Differentiation status	0.301	0.0001
Tumour invasion	0.057	0.350
ER status	0.164	0.007
Her-2 status	-0.162	0.008
Ki67 status	-0.174	0.004

^a^ER, Oestrogen receptor; Her-2, Human epidermal growth factor receptor 2; NDRG2, N-myc downstream-regulated gene 2; TNM, Tumour, node, metastasis. Spearman’s correlation test was used for statistical analyses. *P* < 0.05 was considered statistically significant.

### Correlation of NDRG2 expression with GLUT1 expression in breast carcinoma

Tumour cells depend on glucose as a major substrate for energy production, and many tumour suppressor genes play important roles in the regulation of glucose metabolism [27]. GLUT1 is broadly expressed in the body tissues and is also involved in glucose uptake in the basic state, especially so for tumour glucose metabolism [18]. In our present study, we first investigated this relationship between NDRG2 and GLUT1 expression by IHC and immunoblotting in 30 pairs of breast carcinoma and adjacent normal breast tissue specimens. Positive staining of anti-NDRG2 was found predominantly in the cytoplasm of both normal breast cells and breast cancer cells, but weaker staining for NDRG2 was observed in the cancer specimens compared with normal tissues. Conversely, strong positive staining of GLUT1 was observed in the cytoplasm and cytosolic membrane of tumour cells, and weaker staining was found in noncancerous breast tissue (Figure 1C and Additional file 2: Figures S1 and S2). Furthermore, immunoblotting data revealed that NDRG2 protein expression in cancer samples was much lower than that in the paired adjacent normal tissue. However, GLUT1 expression was higher in cancer tissue than in the paired adjacent normal tissue (Figure 1D and Additional file 2: Figure S3). Indeed, in normal tissue and cancer tissue, the means of immunoreactivity score of NDRG2 IHC staining were 6.03 and 1.97 (Figure 1E), respectively, and the means of immunoreactivity score of GLUT1, IHC Staining were 3.13 and 6.53 (Figure 1F), respectively. Similarly, we identified the degree of correlation between NDRG2 expression and GLUT1 expression in 269 breast cancer specimens. The Spearman correlation analysis indicated that NDRG2 protein expression was inversely correlated with GLUT1 expression (Pearson correlation coefficient *r*_*s*_ = -0.179; *P =* 0.003) (Table 3). The data imply that NDRG2 expression might be involved in the development and progression of breast cancer and that NDRG2 expression is inversely correlated with GLUT1 expression in breast carcinoma.

**Table 3:** **Correlation of NDRG2 expression with GLUT1 expression in 269 breast cancer specimens**^**a**^

**Variable**	***N***	**NDRG2 expression**		***P***^***b***^
		**Low expression, *n *** **(%)**	**High expression, *n *** **(%)**	
GLUT1				0.003^c^
Low expression	130	64 (49.2)	66 (50.8)	
High expression	139	93 (66.9)	46 (33.1)	

^a^GLUT1, Glucose transporter protein 1; NDRG2, N-myc downstream-regulated gene 2. ^b^Spearman correlation test was used for statistical analyses. *P* < 0.05 was considered statistically significant. ^c^Spearman correlation coefficient (*r*_*s*_*)* = -0.179.

### NDRG2 inhibits the growth of breast cancer cells in both high- and low-glucose medium

**Figure 2:**
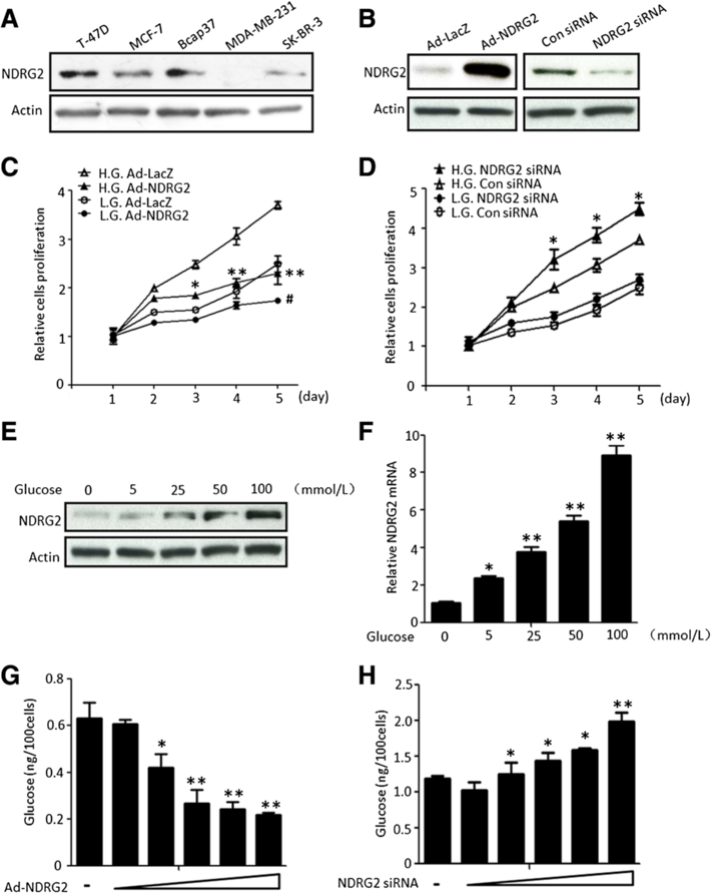
**NDRG2 inhibits cell proliferation and reduces the intracellular glucose levels of breast cancer cells. (A)** T-47D, MCF-7, Bcap37, MDA-MB-231 and SK-BR-3 cells were collected for the extraction of proteins and analysed for N-myc downstream-regulated gene 2 (NDRG2) expression by immunoblotting. **(B)** SK-BR-3 cells with low NDRG2 expression were infected by an adenovirus carrying *NDRG2* (Ad-NDRG2) or negative control LacZ (Ad-LacZ), and T-47D cells with high NDRG2 were transfected with small interfering RNA targeting *NDRG2* (NDRG2 siRNA) or negative control siRNA (Con siRNA). Thereafter proteins were extracted from these cells and analysed by immunoblotting. β-actin was used as a loading control. Before being cultured in 25 mM high-glucose (H.G.) or 5.5 mM low-glucose (L.G.) medium, SK-BR-3 cells were infected by Ad-NDRG2 **(C)** and T-47D cells were transfected by NDRG2 siRNA **(D)**. Cell proliferation was detected by 3-(4,5-dimethylthiazol-2-yl)-2,5-diphenyltetrazolium bromide assay for 1 to 5 days. **(C)** and **(D)** The data presented are means ± SD for three independent experiments; error bars represent SD from 8 replicative wells. **(C)** *P < 0.05 and **P < 0.01 versus H.G. Ad-LacZ. ^#^*P* < 0.05 versus L.G. Ad-LacZ. **(D)** *P < 0.05 versus H.G. Con siRNA. **(E)** and **(F)** SK-BR-3 cells were cultured to glucose medium at concentrations of 0, 5, 25, 50 and 100 mM for 24 hours, and then the protein or mRNA was extracted for analysis by immunoblotting **(E)** or real-time PCR **(F)**. β-actin was used as a loading control. The data presented are means ± SD; error bars represented SD from 3 replicative wells. *P < 0.05 and **P < 0.01 versus control group. **(G)** SK-BR-3 or **(H)** T-47D cells, respectively, were infected with Ad-NDRG2 or transfected NDRG2 siRNA and cultured in a high-glucose medium (25 mM) for 48 hours. Next, cell glucose concentrations were measured. **(G)** and **(H)** The data presented are means ± SD of three independent experiments; error bars represent SD from 8 replicative wells. *P < 0.05 and **P < 0.01 versus control group.

**Figure 3:**
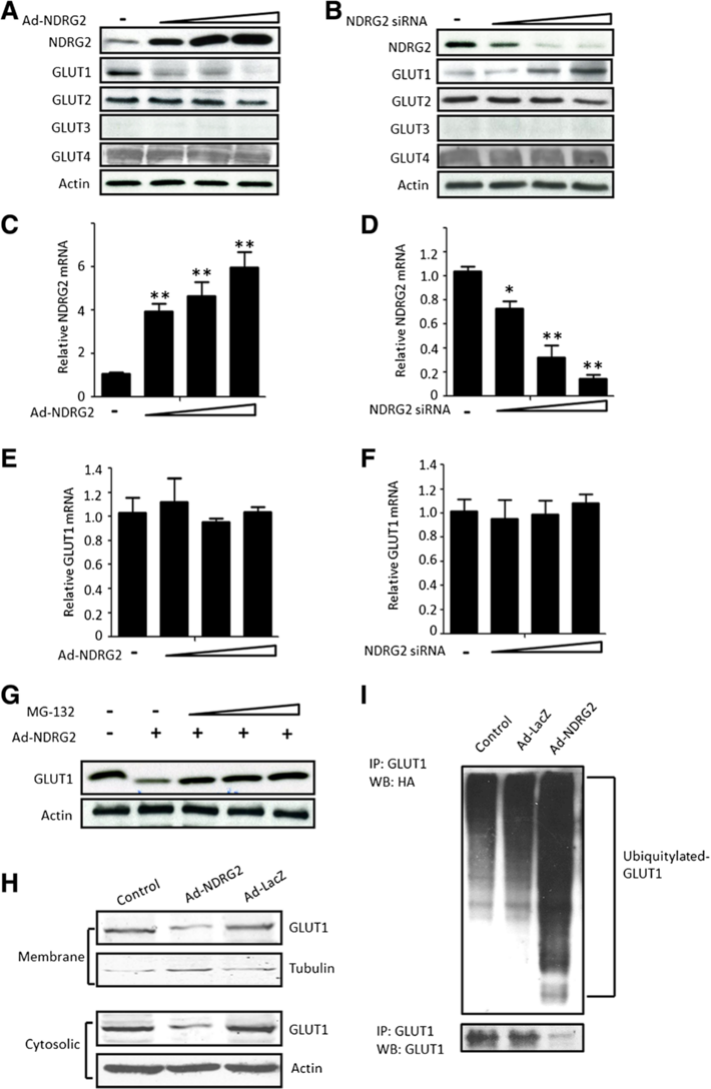
**NDRG2 downregulates GLUT1 by promoting its ubiquitination. (A)**, **(C)** and **(E)** SK-BR-3 cells were infected with an adenovirus carrying N-myc downstream-regulated gene 2 (Ad-NDRG2) at 1, 5 and 10 multiplicity of infection (MOI) or Ad-LacZ for 48 hours. **(B)**, **(D)** and **(F)** T-47D cells were transfected with NDRG2 small interfering RNA (siRNA) 10, 25 and 100 pmol or control siRNA for 48 hours. Next, cell proteins or mRNA were extracted and analysed by immunoblotting **(A)** and **(B)** or by real-time PCR **(C) to (F)**. β-actin was used as a loading control. **(C)** – **(F) **The data presented are the means ± SD of three independent experiments; error bars represent SD from 3 replicative wells. *P < 0.05 and **P < 0.01 versus control group. **(G)** SK-BR-3 cells were infected with 10 MOI Ad-NDRG2 or Ad-LacZ for 48 hours and then treated with 2 μM, 6 μM or 8 μM MG-132 for 4 hours. Next, the protein was extracted and analysed by immunoblotting. **(H)** Cell fractions were prepared from the SK-BR-3 cells infected with 10 MOI Ad-NDRG2 or Ad-LacZ for 48 hours, and the membrane and cytosolic fractions of endogenous glucose transporter 1 (GLUT1) protein were detected. Tubulin and β-actin served as loading controls. **(I)** SK-BR-3 cells were transfected with hemagglutinin (HA)-ubiquitin plasmid for 6 hours and infected with Ad-NDRG2 or Ad-LacZ for another 48 hours. Subsequently, the cell lysates were collected and analysed by immunoprecipitation (IP) and immunoblotting with GLUT1 and HA antibodies. WB, Western blot.

Next, we sought to explore the regulatory mechanism of NDRG2 in tumour glucose metabolism. To investigate the effect of NDRG2 on breast cancer cell proliferation in high-glucose (25 mM) or low-glucose (5.5 mM) medium, we designed the following assays. First, the basic NDRG2 expression in five breast cancer cell lines was examined by immunoblotting. The results showed that the expression of NDRG2 was relatively high in T-47D cells and relatively low in SK-BR-3 cells (Figure 2A). In the following studies, we chose T-47D and SK-BR-3 cells as experimental models. Next, the adenovirus carrying *NDRG2* (Ad-NDRG2) or the siRNA targeting *NDRG2* (NDRG2 siRNA) was applied to upregulate or knock down, respectively, the level of *NDRG2*. After infection by Ad-NDRG2 in SK-BR-3 or transfection by NDRG2 siRNA in T-47D cells, the expression of *NDRG2* was successfully increased or decreased (Figure 2B). Moreover, these effects were more prominent with increasing concentrations of Ad-NDRG2 or NDRG2 siRNA (Figures 3A to 3D). The results of the MTT assays showed that in both high- and low-glucose medium, the overexpression of *NDRG2* inhibited SK-BR-3 cells proliferation (Figure 2C) and silencing *NDRG2* promoted T-47D cell proliferation (Figure 2D). In high-glucose medium, however, the effects of NDRG2 on cell proliferation were more prominent.

### NDRG2 could decrease the intracellular glucose level of breast cancer cells

Given that the antiproliferative effects of NDRG2 on breast cancer cells were more prominent in high-glucose medium, we investigated whether NDRG2 would affect the capacity of these cells to take in glucose. First, the effect of the glucose culture environment on NDRG2 expression was investigated. Immunoblot analysis revealed that, after exposure to different concentrations of glucose medium, the transcription and translation levels of NDRG2 increased with increasing glucose levels (Figures 2E and 2F). In addition, we checked the intracellular glucose content to assess the extent to which NDRG2 contributed to cell glucose uptake. Glucose uptake was decreased with increasing Ad-NDRG2 treatment in SK-BR-3 cells (Figure 2G). T-47D cells were transfected with NDRG2 siRNA at different concentrations, and glucose uptake was significantly augmented with increasing NDRG2 siRNA administration (Figure 2H). These data suggest that a high-glucose microenvironment could promote NDRG2 expression and that NDRG2 could inhibit glucose intake in breast cancer cells.

### NDRG2 negatively regulates GLUT1 levels in breast cancer cells

The transport of glucose across the plasma membrane is the first rate-limiting step for glucose metabolism and is mediated via GLUTs. GLUTs play critical roles in glucose uptake, especially in tumour cells. To investigate whether GLUTs are regulated by NDRG2, GLUT1/2/3/4 protein levels were measured in SK-BR-3 cells infected with Ad-NDRG2 or T-47D cells transfected with NDRG2 siRNA in a high-glucose medium. Immunoblot analysis revealed that GLUT1 protein levels were decreased by increases in Ad-NDRG2-mediated NDRG2 overexpression, but that GLUT1 protein levels were increased with NDRG2 siRNA treatment (Figures 3A and 3B). There were no changes in the intensity of anti-GLUT2 and anti-GLUT4 bands from treated cells compared with the untreated controls. GLUT3 was nearly undetectable in both the SK-BR-3 and T-47D cells (Figures 3A and 3B). However, the data obtained from real-time PCR showed that *GLUT1* transcriptional levels were not significantly changed with NDRG2 up- or downregulation (Figures 3E and 3F). Because the amount of GLUT1 protein can be modified by changes in the rate of synthesis or degradation, we hypothesised that NDRG2 may exert an effect on the degradation of GLUT1. To test this hypothesis, we added the ubiquitin-proteasome inhibitor MG-132 with the Ad-NDRG2-treated SK-BR-3 cells. As shown in Figure 3G, the addition of MG-132 rescued GLUT1 protein levels, indicating that the decrease in GLUT1 in response to NDRG2 overexpression was due to proteasome-dependent degradation. GLUT1 protein is synthesised and degraded in the cytosol, whereas GLUT1 protein enacts its biological function primarily when it is transported to the cell membrane. So, we separated the membrane and cytosolic content of cells to detect GLUT1 protein distribution changes. As shown in Figure 3H, the amount of GLUT1 in the membrane and cytosolic fractions was substantially decreased in NDRG2-overexpressed SK-BR-3 cells. In addition, the immunoprecipitation data revealed that ectopic NDRG2 expression led to an increase in GLUT1 ubiquitination (Figure 3I and Additional file 2: Figure S4). These results suggest that NDRG2 is able to promote GLUT1 ubiquitination and degradation and can lead to decreased GLUT1 protein level.

### NDRG2 interacts with GLUT1

**Figure 4:**
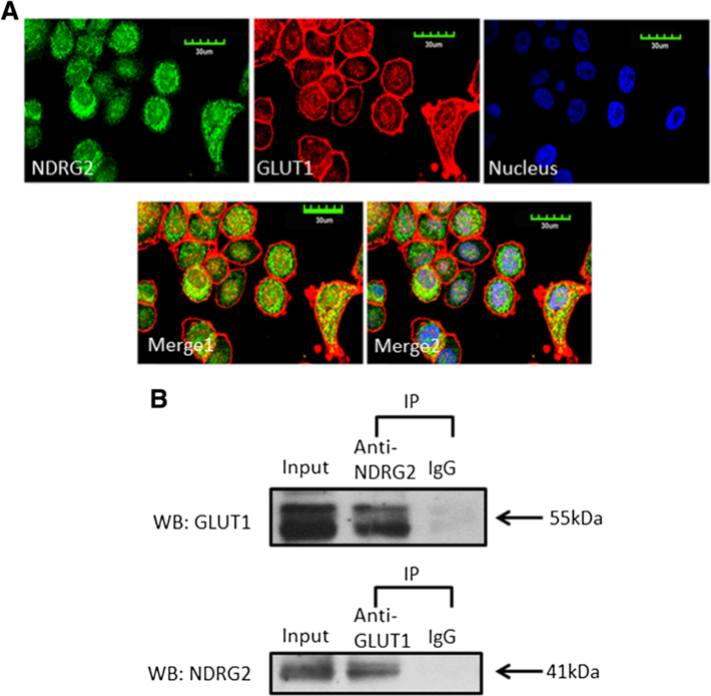
**NDRG2 interacts with GLUT1. (A)** SK-BR-3 cells were fixed and incubated with primary antibodies against N-myc downstream-regulated gene 2 (NDRG2) or glucose transporter 1 (GLUT1) and with fluorescein isothiocyanate or a cyanine 3 secondary antibody. Green fluorescence indicates NDRG2 expression, red fluorescence indicates GLUT1 expression and blue fluorescence indicates nuclear staining. The results of the merged images reveal that NDRG2 and GLUT1 were colocalised in the cytoplasm. **(B)** Immunoprecipitation (IP) assays were performed with whole-cell lysates of SK-BR-3 cells pretreated with protein A–conjugated sepharose beads. Whole-cell lysates were probed for input. The antibodies for immunoprecipitation and Western blot (WB) analyses were carried out as indicated. The locations of various proteins are indicated by arrowheads. IgG, Immunoglobulin G.

Our observation that NDRG2 regulated GLUT1 protein stability prompted us to examine the interaction between NDRG2 and GLUT1 and their subcellular distribution. Confocal microscopy was applied to observe the subcellular localisation of NDRG2 and GLUT1 in SK-BR-3 cells. We found that a portion of endogenous NDRG2 and endogenous GLUT1 was colocalised in the cytoplasmic region of SK-BR-3 cells (Figure 4A). The colocalisation suggests that NDRG2 and GLUT1 may physically interact with each other. To establish whether NDRG2 physically associates with GLUT1, coimmunoprecipitation was performed using lysates prepared from SK-BR-3 cells. A 41-kDa protein that corresponds to NDRG2 was precipitated by anti-GLUT1 antibody and probed by anti-NDRG2 antibody, and a 55-kDa protein corresponding to GLUT1 was precipitated by the anti-NDRG2 antibody and probed by anti-GLUT1 antibody (Figure 4B). We also demonstrated that exogenous GLUT1 coimmunoprecipitated with exogenous NDRG2 in HEK293 cells that were cotransfected with pCMV-flag-NDRG2 and pCMV-eGFP-GLUT1 plasmids and vice versa (Additional file 2: Figure S5). Collectively, the results of these experiments indicated that NDRG2 bound to GLUT1.

### NDRG2 decreases glucose uptake and GLUT1 protein level in subcutaneous xenograft tumours

**Figure 5:**
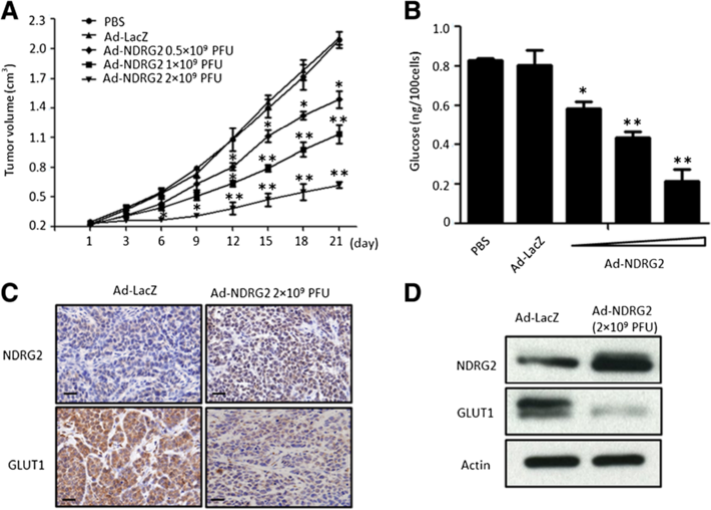
**NDRG2 decreases the glucose uptake and GLUT1 protein levels in SK-BR-3-based subcutaneously xenograft tumours.** The experiments illustrated are described in the Methods section. **(A)** Tumour growth was assessed every 3 days until day 21 treatment by measuring two perpendicular diameters and calculating the volume in cubic centimetres. Ad-LacZ, adenovirus expressing LacZ; Ad-NDRG2, adenovirus expressing NDRG2; PFU, Plaque-forming units. The data presented are means ± SD; error bars represent SD from 6 mice. *P < 0.05 and **P < 0.01 versus phosphate-buffered saline (PBS) or Ad-LacZ. **(B)** Tumour cells were dissociated from xenograft tumours and suspended in PBS after the number of cells was counted. Next, the glucose uptake of cells in each group was detected. The data presented are means ± SD of three independent experiments; error bars represent SD from 6 mice. *P < 0.05 and **P < 0.01 versus PBS or Ad-LacZ. **(C)** Intratumoural protein expression was assessed by N-myc downstream-regulated gene 2 (NDRG2) and glucose transporter 1 (GLUT1) IHC staining. Representative images are shown. Original magnification: 400 x; Scale bars = 50 μm. **(D)** Proteins of the xenograft tumours from each group were extracted and analysed by immunoblotting to quantify NDRG2 and GLUT1 protein changes.

We found that NDRG2 could induce GLUT1 protein degradation to decrease glucose uptake in breast cancer cell lines, but whether this regulatory mechanism also functions in the tumour microenvironment must be studied *in vivo*. We injected Ad-NDRG2 at the concentrations of 0.5, 1 and 2 × 10^9^ PFU or 2 × 10^9^ PFU Ad-LacZ every 3 days into preestablished human SK-BR-3 breast tumours (approximately 200 mm^3^) grown in nude mice. As shown in Figure 5A, the Ad-NDRG2 group that received injections at 2 × 10^9^ PFU achieved a sustained and significant arrest of tumour growth (68% decrease in mean tumour volume on day 21 compared with Ad-LacZ group). The mice were killed at 21 days after the first intratumoural injection, and the tumours were removed for analysis of the glucose uptake and protein levels of NDRG2 and GLUT1. We found that the glucose uptake of tumour cells was inhibited significantly with increased Ad-NDRG2 compared with the Ad-LacZ group (Figure 5B). Consistent with the results of *in vitro* experiments (Figure 3A), increased NDRG2 was correlated with decreased GLUT1 in the xenograft tumours (Figure 5D and Additional file 2: Figure S6). In addition, obvious positive NDRG2 staining was detected by IHC in tumours excised from mice in the 2 × 10^9^ PFU Ad-NDRG2 group. However, GLUT1 staining was weaker in the Ad-NDRG2 group compared with the Ad-LacZ group (Figure 5C). We repeated the xenograft tumour experiments with MDA-MB-231 cells, a highly metastatic cell line, and the results (Additional file 2: Figure S7) were consistent with those of SK-BR-3 cells.

## Discussion

Understanding the mechanisms involved in cancer cell energy metabolism may provide a reasonable interpretation for the function of *NDRG2* as a tumour suppressor. To the best of our knowledge, we report for the first time that NDRG2 participated in cellular glucose uptake by regulating the protein stability of GLUT1. We also found that *NDRG2* adenovirus can be used to treat breast cancer by inhibiting cellular glucose uptake in a nude mouse xenograft model. Consistent with the cell- and animal-based results, a significantly inverse correlation between NDRG2 and GLUT1 expression was observed in clinical breast cancer tissue specimens.

Much of the data obtained from tissue of breast cancer patients presented herein are supportive of previously published work by Oh *et al.*[6]. Although researchers in several studies have reported that NDRG2 inhibited breast cancer cell survival and other malignant activities [5,28-30], the available clinical data before the publication by Oh *et al.* were very limited [6]. Liu *et al.* previously reported that there was a reduction in *NDRG2* mRNA levels in 5 of 21 breast cancer tissue samples tested compared with normal tissues [31]. Anders *et al.* found that NDRG2 protein was reduced in breast cancer tissue based on a slightly larger sample set (*N* = 35) [32]. Recently, the correlation between NDRG2 expression level and clinical meaning was summarized by Oh *et al*. in 189 breast cancer patients who had undergone surgical resection [6]. Similarly to Oh *et al*.’s data set derived from 189 breast carcinoma patients, we show in our present study that, in specimens obtained from 269 breast cancer patients, low NDRG2 expression was associated with advanced TNM stage, high Ki67 and HER2 expression and poor histological differentiation. We also found that breast cancer patients with high NDRG2 expression had longer disease-free survival and better overall survival compared with patients with low NDRG2 expression. However, by Oh *et al*. showed that high NDRG2 expression correlated only with favourable recurrence-free survival, not with overall survival [6]. Further observations are needed to confirm the correlation of NDRG2 and breast carcinoma prognosis. Additionally, in our present study, NDRG2 expression was inversely correlated with GLUT1 expression in patient specimens, which is in agreement with our cell- and animal-based results.

Many tumour suppressor genes play important roles in the regulation of glucose metabolism, in addition to their established roles in cell survival and apoptosis. p53, one of the most highly studied tumour suppressors, which can upregulate NDRG2 expression [4], has been reported to reduce intracellular glucose levels by inhibiting the expression of GLUTs [27]. For example, p53 directly represses the transcriptional activity of *GLUT1* and *GLUT4* gene promoters [33]. In addition, p53 represses *GLUT3* gene expression indirectly by preventing the activation of the inhibitor of the nuclear factor κB pathway [34]. In our present study, we show that NDRG2 could regulate GLUT1 posttranslational modification without affecting other glucose transporters, including GLUT2, GLUT3 and GLUT4. We also found that NDRG2 decreased GLUT1 protein stability by promoting the ubiquitin-mediated protein degradation pathway, whereas the transcription levels of both *GLUT1* and other *GLUT* genes were not affected.

Investigators in previous studies have shown that the expression of *NDRG2* is regulated by some transcription factors, including p53 [4], Myc [35] and Hif-1 [36]. *NDRG2* is a novel p53-inducible target involved in the p53-mediated apoptosis pathway in lung cancer cells [4], and the expression of *NDRG2* was upregulated by Hif-1 in tumour cells under hypoxic conditions [36]. However, the expression of human *NDRG2* is downregulated by Myc via transcriptional repression [35]. c-Myc directly transactivates genes encoding GLUT1 protein and increases glucose uptake in Rat1 fibroblasts [37]. Under hypoxic conditions, a transcription factor complex including Hif-1α was shown to bind the *GLUT1* promoter to upregulate *GLUT1* mRNA expression [38]. Interestingly, among the above-mentioned transcription factors, p53 might promote *NDRG2* expression [4] and inhibit *GLUT1* transcriptional activity [33], and Myc might suppress *NDRG2* expression [35] and transactivate *GLUT1*[37]. *NDRG2* and *GLUT1* were inversely regulated by p53 [4,33] and Myc [35,37], which suggests that *NDRG2* may function as a tumour suppressor by decreasing glucose uptake. Surprisingly, Hif-1 increases the expression of both *NDRG2*[36] and *GLUT1*[38]. We cannot explain why Hif-1 positively regulates both *NDRG2* and *GLUT1* in a manner different from p53 or Myc. We hypothesise that this difference is due to the fact that Hif-1-related experiments were performed under different hypoxic conditions and cell physiological contexts. Whether hypoxia-inducible factors are involved in NDRG2-mediated GLUT1 content and glucose intake regulation in breast cancer needs to be directly determined in future studies.

NDRG2 appears to be broadly involved in stress responses, cell proliferation and cell differentiation [1]. The proteins with which NDRG2 interacts may provide important information contributing to understanding its precise molecular and cellular functions. Our previous study characterised a cell-cycle-dependent transcription factor, MSP58, as a binding partner of NDRG2. NDRG2 may colocalise with MSP58 in the nuclear region of the HeLa cell during cell stress [39]. In another of our previous studies, we found that the β1 subunit of Na^+^/K^+^-ATPase interacted and colocalised with NDRG2 in the perinuclear cytoplasmic region in human salivary cells and that NDRG2 could protect the β1 subunit protein and inhibit its degradation [14]. In that previous study, we detected that NDRG2 bound to and partly colocalised with GLUT1 in the cytoplasmic region of breast cancer cells. We showed that NDRG2 can decrease GLUT1 protein stability and promote the ubiquitination and degradation of GLUT1. Collectively, these experiments imply that NDRG2 might act as some kind of chaperone molecule that is involved in regulating protein stability in different cell physiological contexts. However, the currently available bioinformatics analysis does not indicate any known motif or domain in NDRG2 [40], and, to the best of our knowledge, there is no published literature indicating that NDRG2 is an E3 ubiquitin ligase. Mass spectrometric analysis could be used to screen for the ubiquitin-related proteins that interact with both NDRG2 and GLUT1. However, this hypothesis must be determined directly in future studies.

## Conclusions

To the best of our knowledge, the data produced in our present study provide the first evidence that *NDRG2*, a tumour suppressor, is negatively correlated with GLUT1 expression in breast carcinoma. This correlation is associated with a better prognosis in breast carcinoma patients. We have further demonstrated that NDRG2 plays an important role in tumour cell glucose transport, during which NDRG2 promotes the degradation GLUT1 protein to suppress glucose uptake in breast cancer cells *in vivo* and *in vitro*. Although the contribution of NDRG2 to tumour homeostasis is complicated and not yet fully understood, our findings provide a better understanding of the energy metabolism of tumours. NDRG2 may be viewed as an attractive therapeutic target for breast carcinoma.

## Abbreviations

Akt: Protein kinase B; Cy3: Cyanine 3; DAPI: 4′,6-diamidino-2-phenylindole; DMEM: Dulbecco’s modified Eagle’s medium; ER: Oestrogen receptor; FBS: Foetal bovine serum; FITC: Fluorescein isothiocyanate; GLUT1: Glucose transporter 1; HA: Hemagglutinin; HER2: Human epidermal growth factor receptor 2; Hif-1: Hypoxia-inducible factor 1; IHC: Immunohistochemistry; IP: Immunoprecipitation; MTT: 3-(4,5-dimethylthiazol-2-yl)-2,5-diphenyltetrazolium bromide; NDRG2: N-myc downstream-regulated gene 2; SGK1: Serum- and glucocorticoid-induced kinase 1; siRNA: Small interfering RNA; TNM: Tumour, node, metastasis.

## Competing interests

The authors declare that they have no competing interests.

## Authors’ contributions

All authors conceived of the study and participated in its design. JM, WCL and HG performed most of the experiments. SLi performed xenograft experiments. WC participated in all statistical analyses. XD and SLei provided formalin-fixed, paraffin-embedded, archived patient materials and conducted pathologic reviews and clinical data evaluations. WH performed immunostaining and quantitative analyses. NL and YL interpreted the data and drafted the manuscript. JM, WCL, HG, SLi, WC, XD, SLei, WH, LX and LY revised the manuscript critically. All authors read and approved the final manuscript.

## Supplementary Material

Additional file 1: Table S1 The sequences of small interfering RNA or primers. Click here for file

Additional file 2: Figure S1 Negative control with human breast cancer tissues probed with isotype control IgG. **Figure S2.** NDRG2 is negatively correlated with GLUT1 in breast carcinoma. **Figure S3.** NDRG2 expression inversely correlated with GLUT1 expression in paired tumour and adjacent normal tissues of breast cancer patients. **Figure S4.** NDRG2 downregulates GLUT1 by promoting its ubiquitination. **Figure S5.** The interaction of exogenous NDRG2 and exogenous GLUT1. **Figure S6.** NDRG2 decreases the GLUT1 protein levels in SK-BR-3-based xenograft tumours. **Figure S7.** NDRG2 decreases the glucose uptake and GLUT1 protein levels in MDA-MB-231-based subcutaneously xenograft tumours. Click here for file
