# Supplementary material for: N-myc downstream-regulated gene 2 expression is associated with glucose transport and correlated with prognosis in breast carcinoma
Source: Breast Cancer Res. 2014 Mar 18;16(2):R27. doi: 10.1186/bcr3628 (PMC4053222; doi:10.1186/bcr3628)
Supplement: Additional file 1: Table S1 — The sequences of small interfering RNA or primers. [file bcr3628-S1.doc]

**Table S1. The sequences of siRNA or primers.**

| NDRG2 siRNA | GCACCCAACCTGGATAACATTGAAT |
| --- | --- |
| Control siRNA | GGCCGCAAAGACCTTGTCCTTAGAA |
| GLUT1 (real-time PCR) | Forward: CTTCCTGCTCATCAACCGCA |
|  | Reverse: TGACGATACCGGAGCCAATG |
| NDRG2 (real-time PCR) | Forward: GAGATATGCTCTTAACCACCCG |
|  | Reverse: GCTGCCCAATCCATCCAA |
| β-actin (real-time PCR) | Forward: TGGCACCCAGCACAATGAA |
|  | Reverse: CTAAGTCATAGTCCGCCTAGAAGCA |
