# Supplementary material for: N-myc downstream-regulated gene 2 expression is associated with glucose transport and correlated with prognosis in breast carcinoma
Source: Breast Cancer Res. 2014 Mar 18;16(2):R27. doi: 10.1186/bcr3628 (PMC4053222; doi:10.1186/bcr3628)
Supplement: Additional file 2: Figure S1 — Negative control with human breast cancer tissues probed with isotype control IgG. Figure S2. NDRG2 is negatively correlated with GLUT1 in breast carcinoma. Figure S3. NDRG2 expression inversely correlated with GLUT1 expression in paired tumour and adjacent normal tissues of breast cancer patients. Figure S4. NDRG2 downregulates GLUT1 by promoting its ubiquitination. Figure S5. The interaction of exogenous NDRG2 and exogenous GLUT1. Figure S6. NDRG2 decreases the GLUT1 protein levels in SK-BR-3-based xenograft tumours. Figure S7. NDRG2 decreases the glucose uptake and GLUT1 protein levels in MDA-MB-231-based subcutaneously xenograft tumours. [file bcr3628-S2.doc]

**
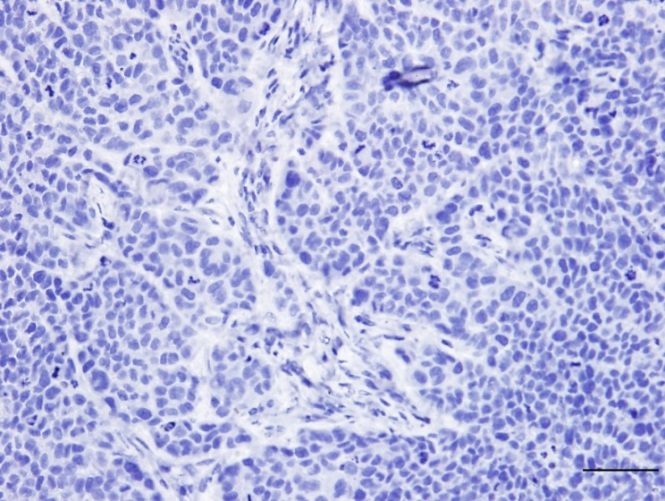
**

**Figure S1 Negative control with human breast cancer tissues probed with isotype control IgG.** Bar = 200 μm


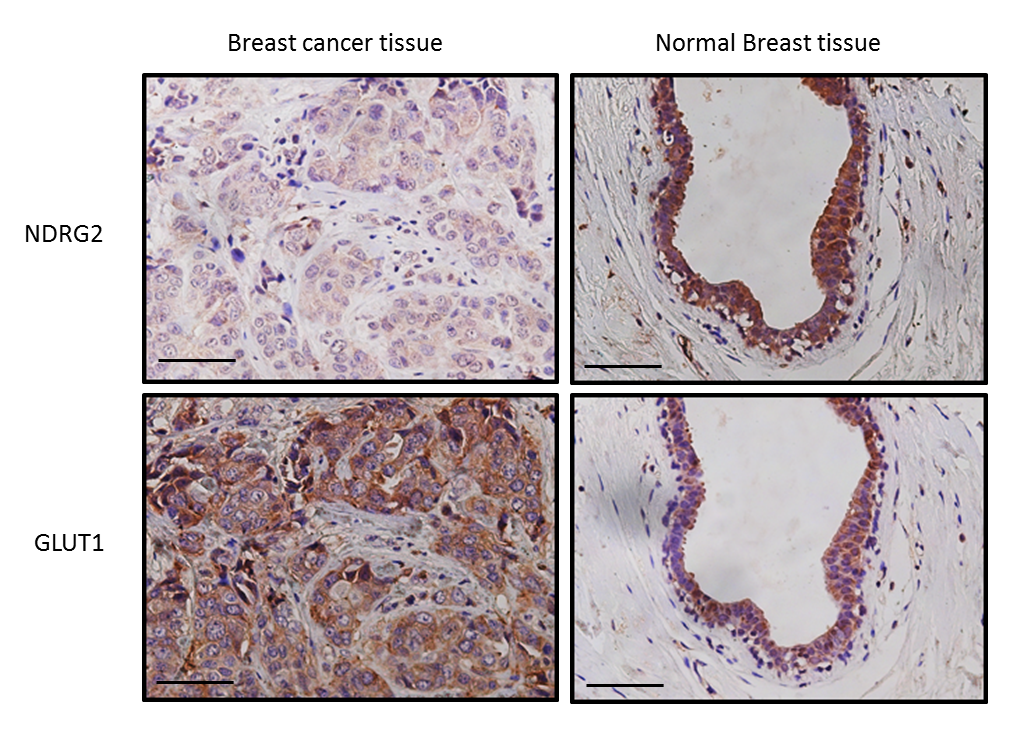


**Figure S2 NDRG2 is negatively correlated with GLUT1 in breast carcinoma.** Serial sections for NDRG2 and GLUT1 immunostainings in breast cancer and normal tissues were analysed. Scale bars = 50 µm.


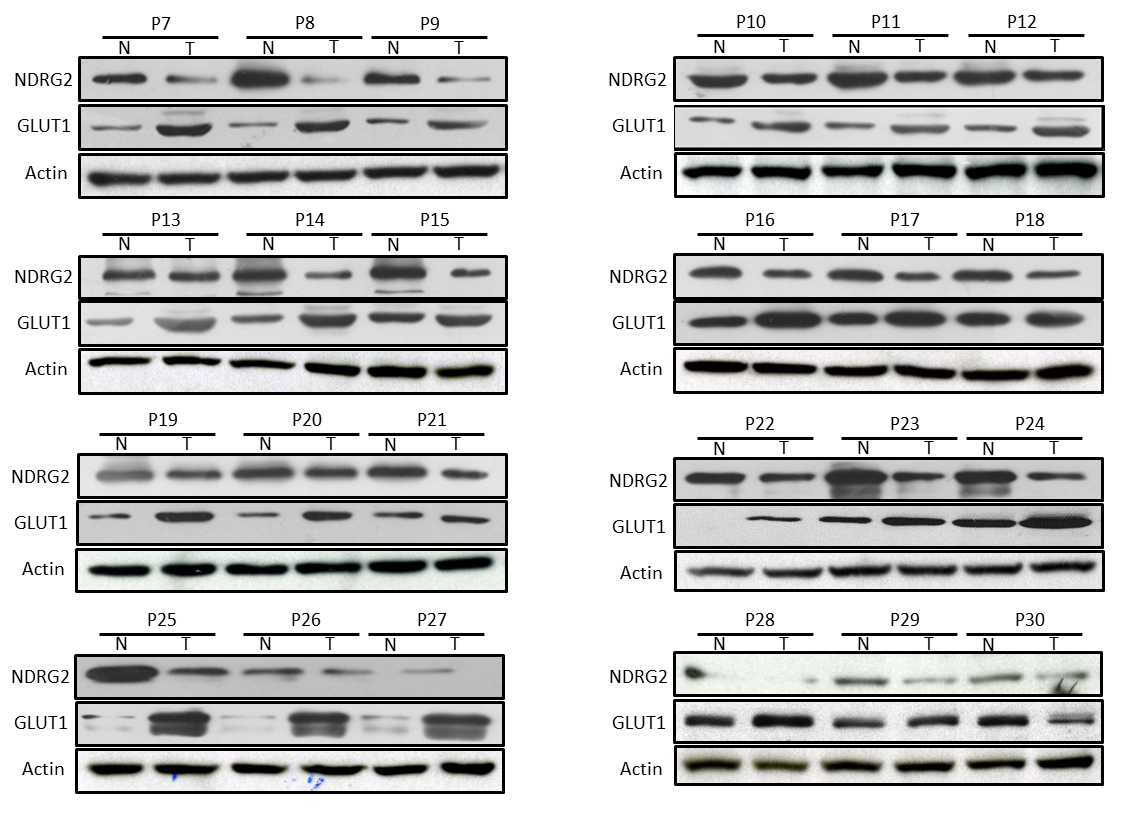


**Figure S3** **NDRG2 expression inversely correlated with GLUT1 expression in paired tumor and adjacent normal tissues of breast cancer patients.** Protein was extracted from paired tissues and subjected to immunoblotting analysis to examine NDRG2 and GLUT1 expression. β-actin served as a loading control. P: patient.

**
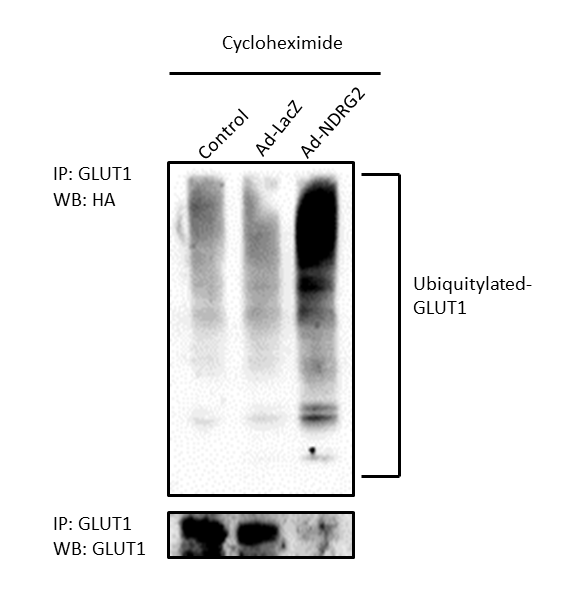
**

**Figure S4 NDRG2 down-regulates GLUT1 by promoting its ubiquitination.** SK-BR-3 cells were transfected with HA-ubiquitin plasmid for 6 h and infected with Ad-NDRG2 or LacZ adenovirus for 48 h, then cells were treated in cycloheximide for 30 min. Subsequently, the cell lysates were collected and subjected to immunoprecipitation and immunoblotting with GLUT1 and HA antibodies. IP, immunoprecipitation; WB, immunoblotting; HA, hemagglutinin.

**
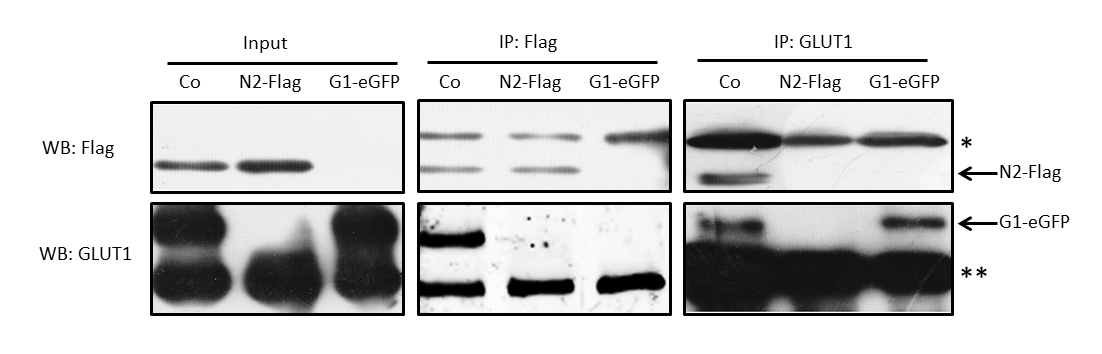
**

**Figure S5 The interaction of exogenous NDRG2 and exogenous GLUT1.** Flag-tagged full-length NDRG2 and/or eGFP-tagged GLUT1 were transfected into HEK293 cells. GLUT1 can be detected in an NDRG2 immunoprecipitate, and conversely, NDRG2 can be detected in the NDRG2 immunoprecipitate when the GLUT1 and NDRG2 are transfected together. Vector transfection alone showed no bands in either direction. Immunoprecipitation antibodies were either anti-GLUT1 or anti-Flag (NDRG2), and the detection antibodies were either anti-GLUT1 or anti-Flag. The location of various proteins is indicated with an arrowhead. N2-Flag, pCMV-flag-NDRG2 transfection alone; G1-eGFP, pCMV-eGFP-GLUT1 transfection alone; Co, co-transfection with both plasmids; WB, immunoblotting; IP, immunoprecipitation. * indicates Ig heavy chain; ** indicates endogenous GLUT1.

**
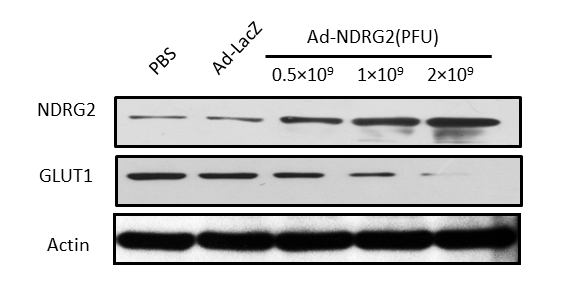
**

**Figure S6 NDRG2 decreases the GLUT1 protein levels in SK-BR-3-based xenograft tumours.** The proteins of the xenograft tumours from each group were extracted and subjected to immunoblotting analysis to examine NDRG2 or GLUT1 protein changes.


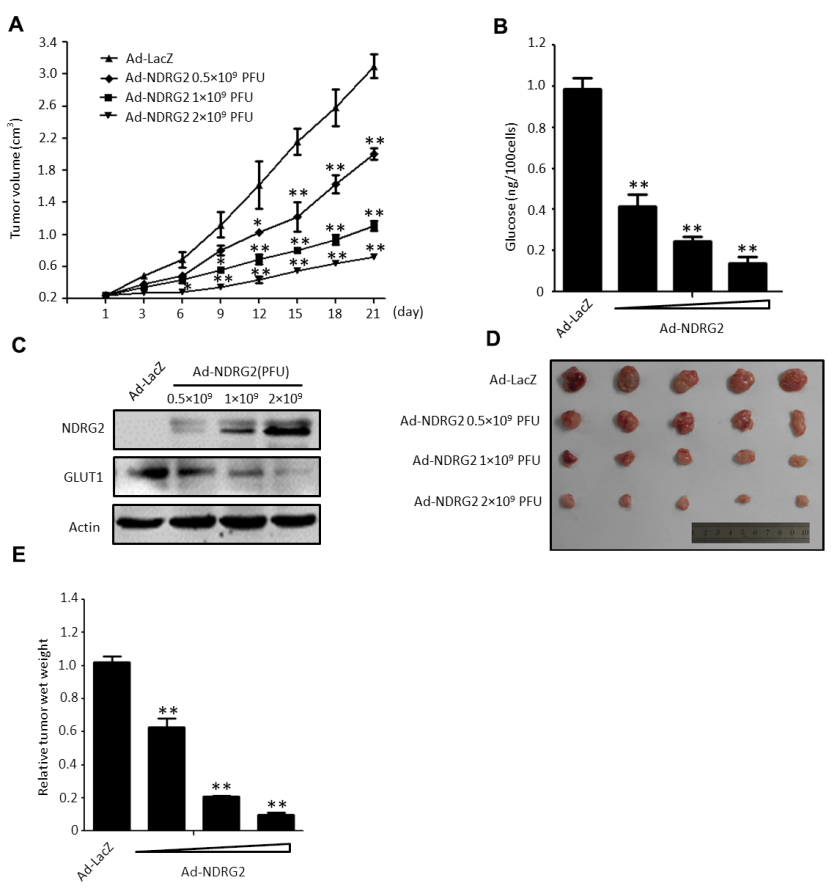


**Figure S7 NDRG2 decreases the glucose uptake and GLUT1 protein levels in MDA-MB-231-based subcutaneously xenograft tumours.** These experiments were described in the ‘‘Materials and Methods’’. (**A**) The tumour growth was assessed every 3 days until day 21 treatment by measuring 2 perpendicular diameters and calculating the volume in mm^3^. The data are presented as the means ± S.D. (n=5), * *p* < 0.05; ** *p* < 0.01 versus Ad-LacZ. (**B**) Tumour cells were dissociated from xenograft tumours and suspended in PBS after counting the number of cells. Then, the glucose uptake of cells from each group was detected. The data are presented as the means ± S.D. of 3 independent experiments. ** *p* < 0.01 versus Ad-LacZ. (**C**) Protein of the xenograft tumours from each group were extracted and subjected to immunoblotting analysis to quantify NDRG2 or GLUT1 protein changes. (**D**) The image of individual tumours. (**E**) Relative final tumour wet weights were measured. The data are presented as the means ± S.D. (n=5), ** *p* < 0.01 versus Ad-LacZ.
